# A pushing-grasping collaborative method based on deep Q-network algorithm in dual viewpoints

**DOI:** 10.1038/s41598-022-07900-2

**Published:** 2022-03-10

**Authors:** Gang Peng, Jinhu Liao, Shangbin Guan, Jin Yang, Xinde Li

**Affiliations:** 1 School of Artificial Intelligence and Automation, Huazhong University of Science and Technology, Wuhan, 430074 China; 2 Key Laboratory of Image Processing and Intelligent Control, Ministry of Education, Huazhong University of Science and Technology, Wuhan, 430074 China; 3 School of Automation, Southeast University, Nanjing, 214135 China

**Keywords:** Computer science, Information technology, Software

## Abstract

In the field of intelligent manufacturing, robot grasping and sorting is important content. However, there are some disadvantages in the traditional single-view-based manipulator grasping methods by using a 2D camera, where the efficiency and the accuracy of grasping are both low when facing the scene of stacking and occlusion for the reason that there is information missing by single-view 2D camera-based methods while acquiring scene information, and the methods of grasping only can’t change the difficult-to-grasp scene which is stack and occluded. Regarding the issue above, a pushing-grasping collaborative method based on the deep Q-network in dual viewpoints is proposed in this paper. This method in this paper adopts an improved deep Q-network algorithm, with an RGB-D camera to obtain the information of objects’ RGB images and point clouds from two viewpoints, which solved the problem of lack of information missing. What’s more, it combines the pushing and grasping actions with the deep Q-network, which make it have the ability of active exploration, so that the trained manipulator can make the scenes less stacking and occlusion, and with the help of that, it can perform well in more complicated grasping scenes. In addition, we improved the reward function of the deep Q-network and propose the piecewise reward function to speed up the convergence of the deep Q-network. We trained different models and tried different methods in the V-REP simulation environment, and it drew a conclusion that the method proposed in this paper converges quickly and the success rate of grasping objects in unstructured scenes raises up to 83.5%. Besides, it shows the generalization ability and well performance when novel objects appear in the scenes that the manipulator has never grasped before.

## Introduction

As of the main problems in the field of robotic operation and intelligent manufacturing, grasping is the main measures for robots to interact with the real world to complete operational tasks (^1^, such as sorting, assembly, etc.). The traditional 2D camera-based robot grasping methods perform well in grasping with high success rate when facing simple scenes^2^ uses thresholding and polygon encircling algorithms to locate the fruit to be grasped, and the grasping success rate reaches 85%^3^ proposed the improved HOG (Histogram of Oriented Gradient) which is used to identify the industrial components for grasping, and the success rate of grasping components came up to 92%. A method is proposed by Zhao Yuansen aiming at grasping tomatoes, combining with two color spaces’ images, using thresholding to locate and recognize the targets, and the success rate of grasping came up to 93%. However, in actual grasping applications, there are a large number of gathering and covering objects in grasping scene. Since 2D cameras cannot obtain information of height, it is difficult for robotic grasping system based on 2D cameras to grasp gathering and covering objects.

Deep reinforcement learning combines the perception ability of deep learning and the decision-making ability of reinforcement learning^4,5^, so that many grasping methods based on deep reinforcement learning emerged, and the depth camera can obtain deep information, which further improved the cognition level of the robot. A method proposed by^6^ of deep reinforcement learning in robotic grasping work was proposed for the first time, and it pointed out that the deep reinforcement learning algorithm model trained in a simulation environment can be applied to the grasping environment in reality through less training steps of grasping real objects. A method of knowledge-induced deep Q-network (DQN) was proposed by^7^. The network integrates perceptual information into DQN to provide prior knowledge for network training, so that the trained model has better generalization ability than traditional DQN, and the manipulator can better grasp unknown objects which do not exist in training. In the work of^8^, taking Actor-Critic in deep reinforcement learning as the main body, with redesigning the reward function, a reward function designing method based on objects structure matching was proposed. The improved algorithm converges faster with higher success rate of grasping. In the work of^9^, it improves the experience playback mechanism of the depth deterministic strategy gradient algorithm proposed by Google, which not only improves the performance of the algorithm, but also reduces the use of storage space and improves the efficiency of the manipulator to grasp objects. However, there are still problems in the current grasping methods based on deep reinforcement learning such as insufficient information utilization, network convergence slowly, and low success rate of grasping. In the two works of^10^ and^11^, a method of using the affordance map to represent the grasp target by analyzing the whole scenes is proposed, which greatly improves the accuracy of the robot grasping. However, because the environment is usually complex and unstructured, sometimes the grasping position indicated in the affordance map is difficult for manipulators to grasp. In the work of^12^, a method of active affordance exploration for robot grasping is proposed but it didn’t provide the design guidelines of the reward. In the work of^13^, an active exploration algorithm which leverages the deep Q-Network (DQN) is proposed to facilitate the robot to actively explore the environment until a good affordance map is generated, but it just process images from a single viewpoint, so there are inevitably problems of information missing. In summary, the main contributions of this paper are:

The grasping method for manipulators above can hardly perform well in unstructured scenes that appear as gathering and covering, for the reason that it can’t recognize objects accurately in cluster scenes from a single viewpoint and the manipulators can’t make the environment better for grasping. In this case, a pushing-grasping collaborative method based on the deep Q-network in dual viewpoints was proposed to solve the problems of the information missing in a single viewpoint and the limitations in grasping action only. What’s more, we improved the reward function of the deep Q-network and propose the piecewise reward function to speed up the convergence of the deep Q-network to solve the problem of divergence and non-convergence caused by sparse reward. We train the model of robot grasping iteratively. The conclusions show that the trained model gives the best actions manipulator takes, which also has a high success rate of grasping and generalization ability for grasping unknown objects which had never been grasped before.

The rest of this study is organized as follows: The principle and implementation process of the method proposed are presented in “Related work” section. In “Design of pushing-grasping method” section, it shows the results and analysis of experiment in simulation environment. Last but not least, it draws a conclusion in “Results and analysis of experiment in simulation environment” section.

## Related work

The deep reinforcement learning method combines deep learning and reinforcement learning, uses deep learning to automatically learn the abstract features of large-scale input data, and uses the reinforcement learning part to make decisions. The trial-feedback mechanism is used to make the robotic arm continuously interact with the environment, and gradually obtain better action decisions during the interaction with the dynamic environment, and then complete tasks in an unstructured environment^14^. The current research directions mainly focus on deep reinforcement learning algorithms based on value functions and reinforcement learning algorithms based on policy gradients.

In terms of deep reinforcement learning algorithms based on value functions, Zhang et al.^7^ used the deep Q learning algorithm to establish and develop a robotic arm learning system that uses a camera as input to enable a 3-DOF robot to achieve a target point The task that arrives can be applied to the sorting of items at the same time. Bai et al.^15^ proposed a hybrid algorithm DMQDN based on a competitive architecture and dual-depth Q-learning, designed a discrete robotic arm motion space, and used visual information from multiple perspectives to be used in the multi-task learning process of the robotic arm. James et al.^16^ used deep Q-learning and 3D simulation to train a 7-DOF robot arm without any prior knowledge, so that a robot arm can complete the end-to-end grasping control task. Guo Xian of University of Science and Technology Beijing^17^ proposed two improved algorithms based on deep Q learning, namely guided deep Q learning algorithm and recursive deep Q learning algorithm, to complete the grasping tasks of robotic arms in sorting tasks. Gu et al.^18^^6^ proposed a normalized advantage function (NAF) algorithm based on the deep Q learning algorithm, which was applied to robot clusters and evaluated some complex operation tasks (Including door opening and pick-and-place) advantages.

There are a great number of reinforcement learning algorithms research based on policy gradient. Lillicrap et al.^19^ used the idea of deep Q learning algorithm to set the current network and target network according to specific scenarios, and compared the deterministic policy gradient (DPG) method. Redesigned and proposed a Deep Deterministic Policy Gradient (DDPG) algorithm based on AC (Actor-Critic) framework. This algorithm can be used to solve the deep reinforcement learning problem in the continuous action space, and it can solve many physical tasks, such as controlling the inverted pendulum, end-to-end operation (arrival, handling) of the robotic arm, car driving, etc. Therefore, the algorithm is widely used Development and improvement. Li^20^ proposed a fault-tolerant control method for a manipulator based on DDPG, so that the manipulator can still effectively complete the task of reaching the target point when a single joint fails. In addition, Heess et al.^21^ proposed the Stochastic Value Gradient (SVG) method, which aims to be applied to the control of the continuous motion of the robotic arm. This method is used to complete the task of screwing caps similar to the sorting task. Levine et al.^22^ proposed a robotic arm grasping method that does not require hand-eye calibration, and trains the parameters of the neural network through a large number of operations, and determines the actions to be executed according to the establishment of the grasping prediction network to complete the grasping task. Finn et al.^23^ proposed a model-based reinforcement learning method. By building a deep prediction model and a strategy search algorithm to train unlabeled training data, it can control the robotic arm without calibration and manual intervention. Push different items to the designated target location. Haarnoja et al.^24^ proposed a gradient algorithm based on deterministic strategy, using the maximum entropy strategy to apply to the actual robot control tasks, carrying out operations such as handling and placing, so that the robotic arm can complete the multi-step operation task of stacking wood.

The DQN algorithm used in this article belongs to a deep reinforcement learning algorithm based on a value function.

## Design of pushing-grasping method

When the robot is grasping objects in current scene, the state of scene after grasping depends on the current state of the scene and the actions taken by the manipulator. The grasping process can be described by Markov decision process (MDP)^25^. Markov decision process contains 5 elements: state set S, action set A, state transition probability matrix P, reward set R and the discount factor $\gamma$. The grasping process of the manipulator can be described as in the state $s_t$. According to the strategy $\pi$, the manipulator takes action $a_t$ with probability P($s_{t+1}$ | $s_t$,$a_t$ ) to act on the scene so that it changes into the new state $s_{t+1}$, and get the reward $r_t$. The goal of reinforcement learning for manipulators is to find an optimal strategy $G_t$ and the grasping action of manipulators can obtain the maximum return $G_t$ cumulatively, where $\gamma$ is a discount factor which aims to reduce the influences of current rewards acting on future behavior.

1 $$\begin{aligned} { G_t = R_{t+1} +\gamma R_{t+2} +\gamma ^{2} R_{t+3} +...=\sum _{k=0}^T \gamma ^{k} R_{t+1+k} } \end{aligned}$$

According to Markov decision process, we can know that reinforcement learning needs to store the mapping relations between the state and the action, which is called the learning strategy. The mapping relations are generally stored in a table. For large-scale problems, the tables can hardly be maintained and stored in memory. Therefore, this paper adopts a method of deep reinforcement learning based on value function-deep Q network^26,27^. DQN takes the feature vector $\phi (s)$ of state s as input, and outputs the state-action value function *Q*(*s*, *a*) which corresponds to each action through network processing. DQN adopts an approximate description of the value function, so it evolves into Regression problem, called function fitting:

2 $$\begin{aligned} { Q(s, a; \theta )\approx Q^* (s, a) } \end{aligned}$$

Where $\theta$ represents model parameters. In order to speed up convergence of the network, we designed two convolutional neural networks: current value network function Q(s, a; $\theta$)and target value network functionQ(s, a; $\theta ^{-}$). The parameters of the current value network will be updated with the iteration process, and the target value network will calculate the Q value along the iteration process, but the network parameters will be fixed to a certain number of iteration steps. After certain steps, the parameters of the current value network are copied and updated, so the optimization goal of model is:

3 $$\begin{aligned} C=R+\gamma max _{a^{'}} Q(s^{'},a^{'};\theta ^{-}) \end{aligned}$$

Therefore, by minimizing the square error of the current value function and the target value function, the network parameters can be updated.

4 $$\begin{aligned} L(\theta )=E[(R+\gamma max_{a^{'}} Q(s^{'},a^{'};\theta ^{-} )-Q(s,a;\theta ))^{2} ] \end{aligned}$$

A. Description of state set

**Figure 1:**
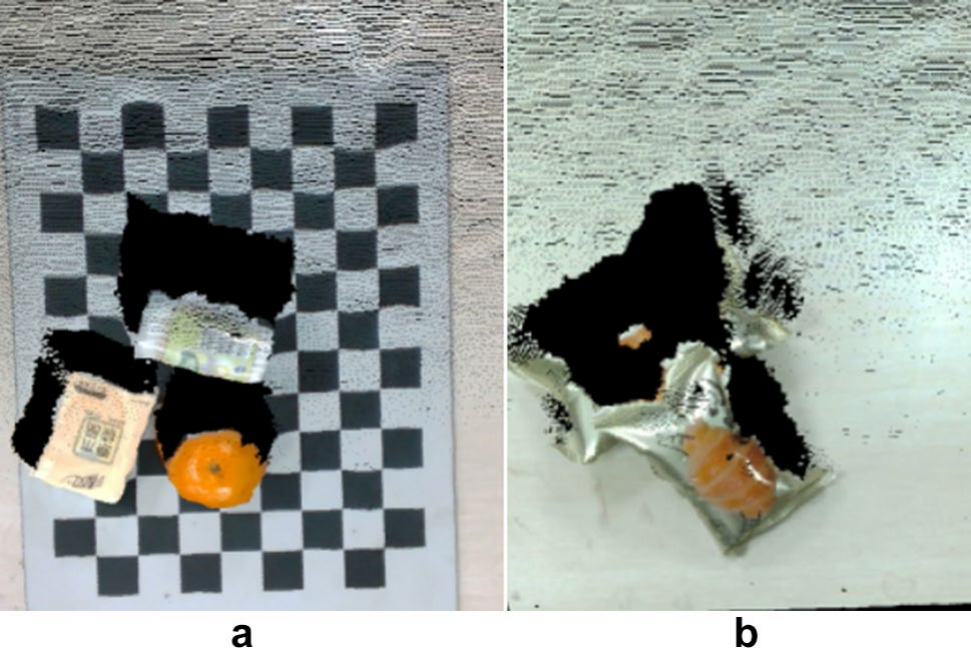
Information missing in single viewpoint.

In this paper, we use RGB-D cameras to obtain the point cloud of objects, but the point cloud image cannot be put directly into the DQN as input, so this paper takes the RGB top view of point cloud in current scene before each action as a state $s_t$ as the state input of the DQN. But for gathering objects, if the camera is still above the grasping area and installed vertically downwards, it will make a difference to plans of grasping. Therefore, a method with certain angle installation which is placed outside the grasping area is adopted, but there will be information missing when the top view is obtained under a single angle view, as shown in Fig. 1. This paper uses two RGB-D cameras to capture from two viewpoints so that complete object information is guaranteed. The pixel resolution of the RGB top view obtained is 224 × 224.

B. Description of action space

For the purpose of adapting complex grasping scenes, this paper introduces a manipulator pushing based on the grasping. For the reasons that pushing can disrupt the original arrangement of the objects, which makes it more convenient for manipulator to grasp. Two pieces of information need to be obtained when the manipulator takes action of grasping or pushing, namely target position (x,y,z) that the gripper needs to move to and the postures of the gripper. The target position (x,y) can be learned by DQN, and the height z can be obtained from depth maps; for the postures of the end manipulator, this paper adopts a vertical downward grasping method, so there is only a rotation angle $\theta$ along z-axis in the postures of the end manipulator, and the manipulator can push along the direction of an angle $\alpha$ while it is pushing.

When the manipulator is pushing or grasping objects, it is not necessary for the end rotation angle and the pushing angle of the manipulator to be an accurate angle. Therefore, we discretize the angles above and divides 360° into 16 equal parts, which are defined by 22.5° as a grasping or pushing direction. Therefore, the action space can be described as:

Pushing action: taking coordinates (x,y) learned by the DQN as the starting position, the end of the manipulator pushes the object in one of the 16 directions set, and the pushing distance is half the length of the longest side of the objects, which is set to 5cm in this paper.

Grasping action: Taking coordinates (x,y) learned by the DQN as the target position of the center movement of the gripper at the end of the manipulator, which is rotated to one of the 16 directions above to grasp the objects.

It can be seen from the state set that the descriptions of state use a top view, while the visual calibration uses an oblique view as usual. While manipulator taking actions to push or grasp, we need to determine the conversion between the two images, and the conversion relationship can be determined by viewpoint transformation.

C. Design of value network

Due to the recognition of images, in this paper, we model the Q function as two Fully Convolutional Networks (FCN), which was proposed by^28^, one of which is used to select the pushing action and the other is used to select the grasping action. This two networks have the same structure: each network contains a pre-trained DenseNet121(^29^), and then is channel-cascaded with two 1 × 1 convolutional layers, and each convolutional layer has a ReLU activation function and batch normalization, and finally bilinear upsampling, each network will capture the RGB top view of point cloud of the first two viewpoint scenes as input.

**Figure 2:**
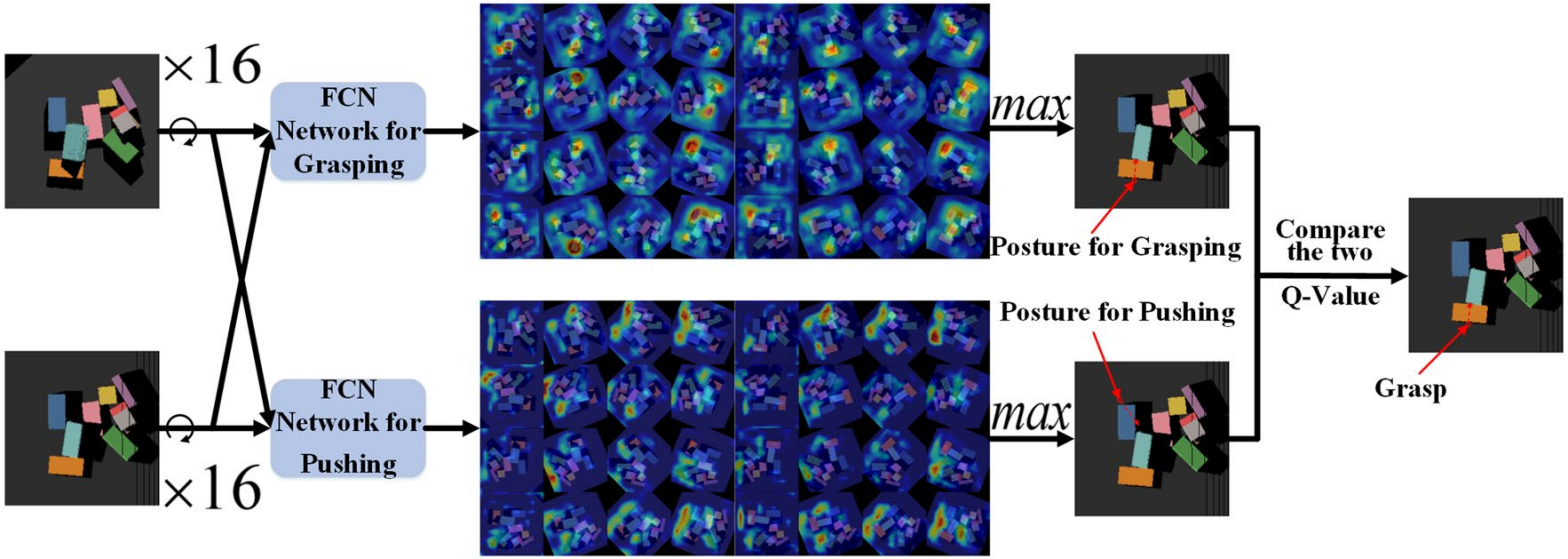
Process of the DQN algorithm.

In order to obtain the suitable angle of pushing or grasping, we rotate the RGB top view 15 times as the input of FCN. That is, for each FCN network, 16 × 2 pictures will be set as input, and output will be the same size and number of heat maps as input images. Each pixel of pictures represents an independent Q value prediction. Sorting the Q values of the pixels in the output heat maps of the two networks, we can respectively obtain the heat maps’ largest Q value in the pushing network and grasping network, and we can select the Q value in the two heat maps who is larger as the final network output (Fig. 2). The final selected pixel and direction are given by the pixel corresponding to the maximum Q value in the heat maps which the network finally output, and the rotation angle corresponding to the heat maps. The selected action is given by the network corresponding to the heat map.

D. Reward configuration

The traditional reward is set as a reward R=1 for a successful grasping. If the total change of the difference between height maps exceeds a certain threshold $\tau$, a reward R = 0.5 is given. Such a reward is too sparse, which will lead to non-convergence and slow convergence in model training. In order to deal with the problems, a piecewise reward strategy instead of a single reward is proposed in this paper. The piecewise reward function is defined as:

5 $$\begin{aligned} { R=\left\{ \begin{array}{rcl} 1 &{} &{} {grasp\ successfully}\\ -1 &{} &{} {grasp\ failed}\\ 0.3 &{} &{} {pixel\ change\ \tau (10\% \sim 24\%)}\\ 0.5 &{} &{} {pixel\ change\ \tau (24\% \sim 40\%)}\\ 0.7 &{} &{} {pixel\ change\ \tau (40\% \sim 100\%)}\\ -0.1 &{} &{} {otherwise} \end{array} \right. } \end{aligned}$$

where R is the reward of taking the action chosen by the decision making of the deep Q-learning network. If grasp successfully, the reward R = 1 is given, if grasp failed, the reward R = − 1 is given; if the rate of pixel change $\tau$ of the scene after pushing is 10% 24%, the reward R = 0.3 is given; If the rate of pixel change $\tau$ is between 24% 40%, the reward R=0.5 is given; if the rate of change $\tau$ is 40% 100%, the reward R = 0.7 is given; otherwise, the reward R = − 0.1 is given.

### Consent to participate

All authors confirm that they are involved to this study.

### Consent for publication

The authors confirm that the manuscript has been read and approved by all named authors.

## Results and analysis of experiment in simulation environment

**Figure 3:**
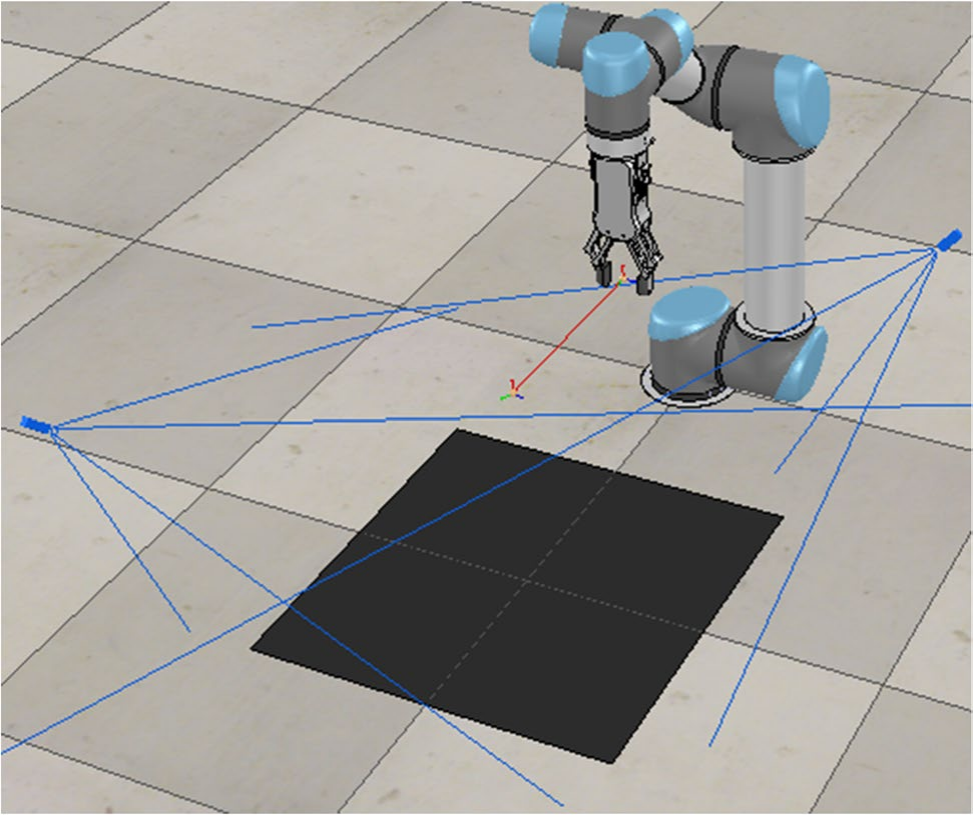
Simulation environment of grasping.

In order to verify that the DQN algorithm after adding pushing action and dual viewpoints has a higher success rate of grasping and generalization ability, a comparative experiment of the two algorithms is carried out on the simulation platform for the grasping in the covering scene. The hardware used is the Intel i7-8700k as CPU and Nvidia GTX 1080Ti 16GB as GPU. V-REP^30^ is used for training and verification under the environment of Ubuntu 16.04. As shown in Fig. 3, the methods above are used to learn how to grasp objects in the area.

Stochastic gradient descent is used to train the FCN network during training. In the experiment, the learning rate is set to 10^−4^, and the weight decay is set to 2^−5^. The exploration strategy adopts the $\epsilon$-greedy algorithm. The initial $\epsilon$ is set to 0.65, the annealing is set to 0.1 after training, and the discount factor $\gamma$ is set to 0.6.

Compared with the grasping only strategy, the DQN algorithm based on dual viewpoints in this paper improves in 3 aspects: ➀ The dual viewpoints are used to obtain the objects’ information in the area to be grasped, avoiding missing information in single viewpoint; ➁ A novel pushing action is introduced, which makes up for the disadvantage of the method with grasping only which can not change the complicated environment like stack and occlusion; ➂ Adopt piecewise reward strategy, which solve the problems of slow convergence caused by single reward. In order to verify the impact of the improvement above on grasping performance, we design a series of comparative experiment for verification in this section.

A. Comparative experiment

In this experiment, the pushing and grasping strategy based on DQN and dual viewpoints was compared with three methods of removing the improvement aspects to verify the influence of each improvement aspect on the performance of the algorithm. Zhang et al.^7^ used DQN to establish a learning system that uses single viewpoint as input to enable a 3-DOF robot to achieve a target point, which can only get the incomplete information obviously. Bai et al.^15^ proposed a hybrid algorithm DMQDN based on dual-depth Q-learning, which designed a motion space, and it got visual information from multiple perspectives, but it takes the method using grasp only without pushing so it can not change the complex environment for grasping. Zeng et al.^10^ proposed a method of using the affordance map to represent the grasp target by analyzing the whole scenes is proposed, which greatly improves the accuracy of the robot grasping. However, they train the network only use single reward which make the trainning more slowly.

**Figure 4:**
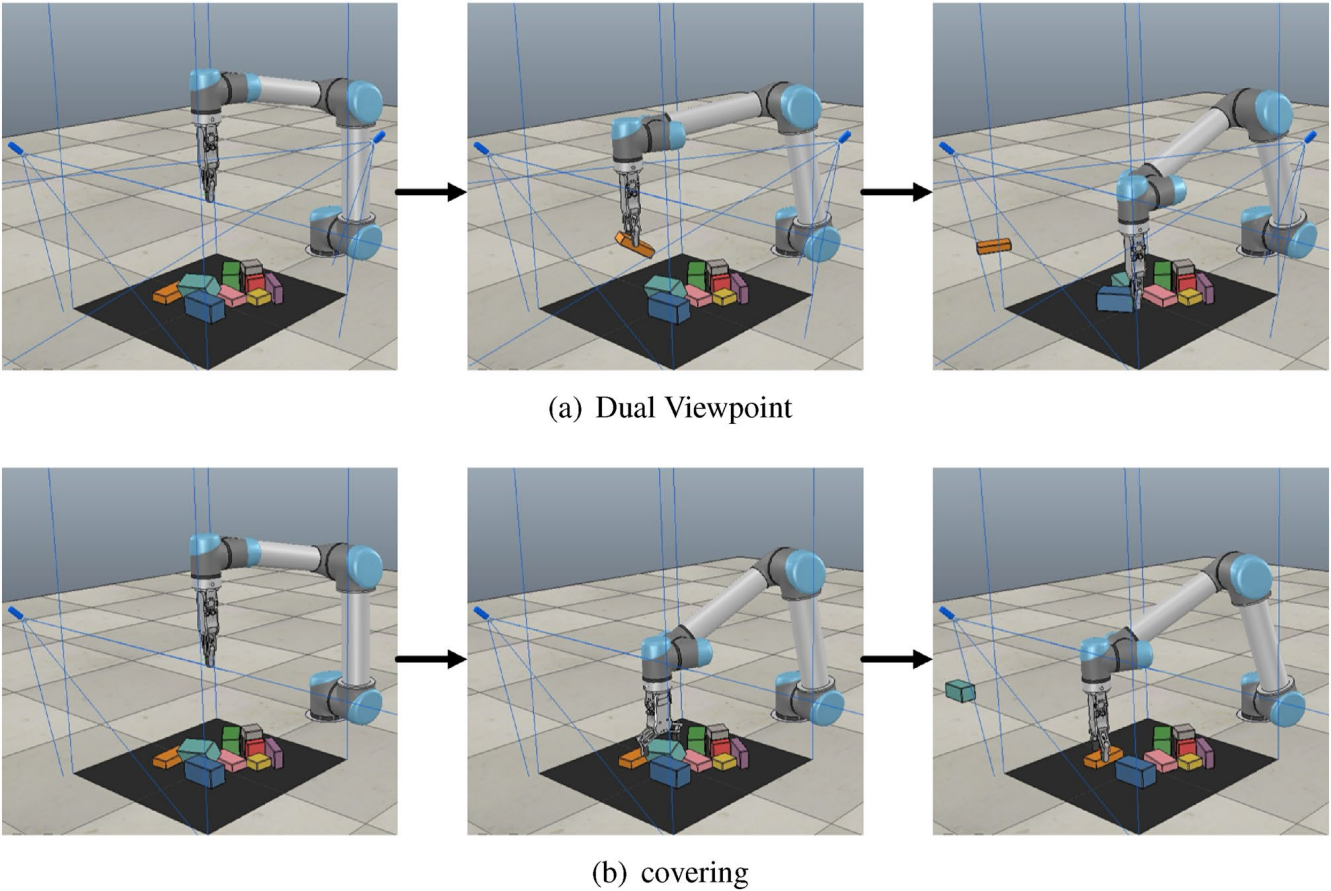
Information missing in single viewpoint.

The training scene is set as follows: select 10 objects randomly arranged and put them in the grasping area. Manipulator learns how to grasp the objects in the area through trial and error, until the objects in the work space are grasped completely or out of the view, and then select 10 objects randomly again and place them in the grasping area. Every 100 objects grasped is a group of training. After one group of training, the next group of training starts. The training process is shown in Fig. 4.

**Figure 5:**
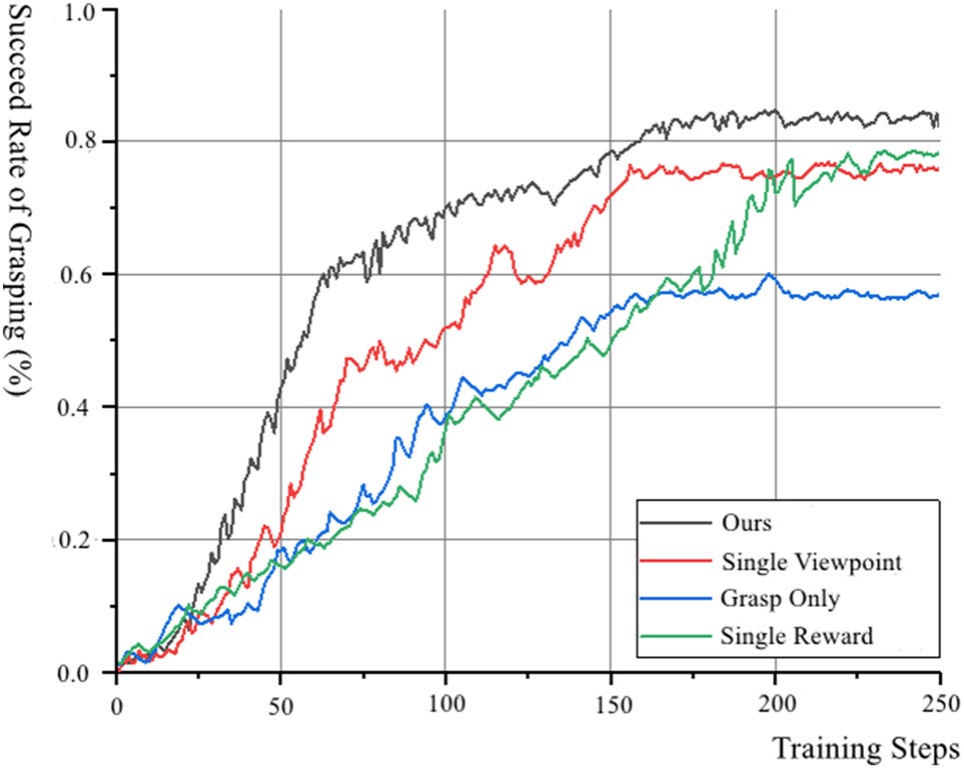
Success rate of different methods to grasp.

The evaluation of performance is mainly to evaluate the success rate of grasping and the convergence speed of the model. The success rate of grasping mainly refers to the ratio of the number of objects grasped successfully to the number of objects to be grasped. Compared with the method with single viewpoint^7^, the method which use grasp only without pushing action^15^ and the method with single reward^10^, the comparison between the three methods above and ours is as Fig. 5 shown.

B. Tests of model’s generalization ability

**Figure 6:**
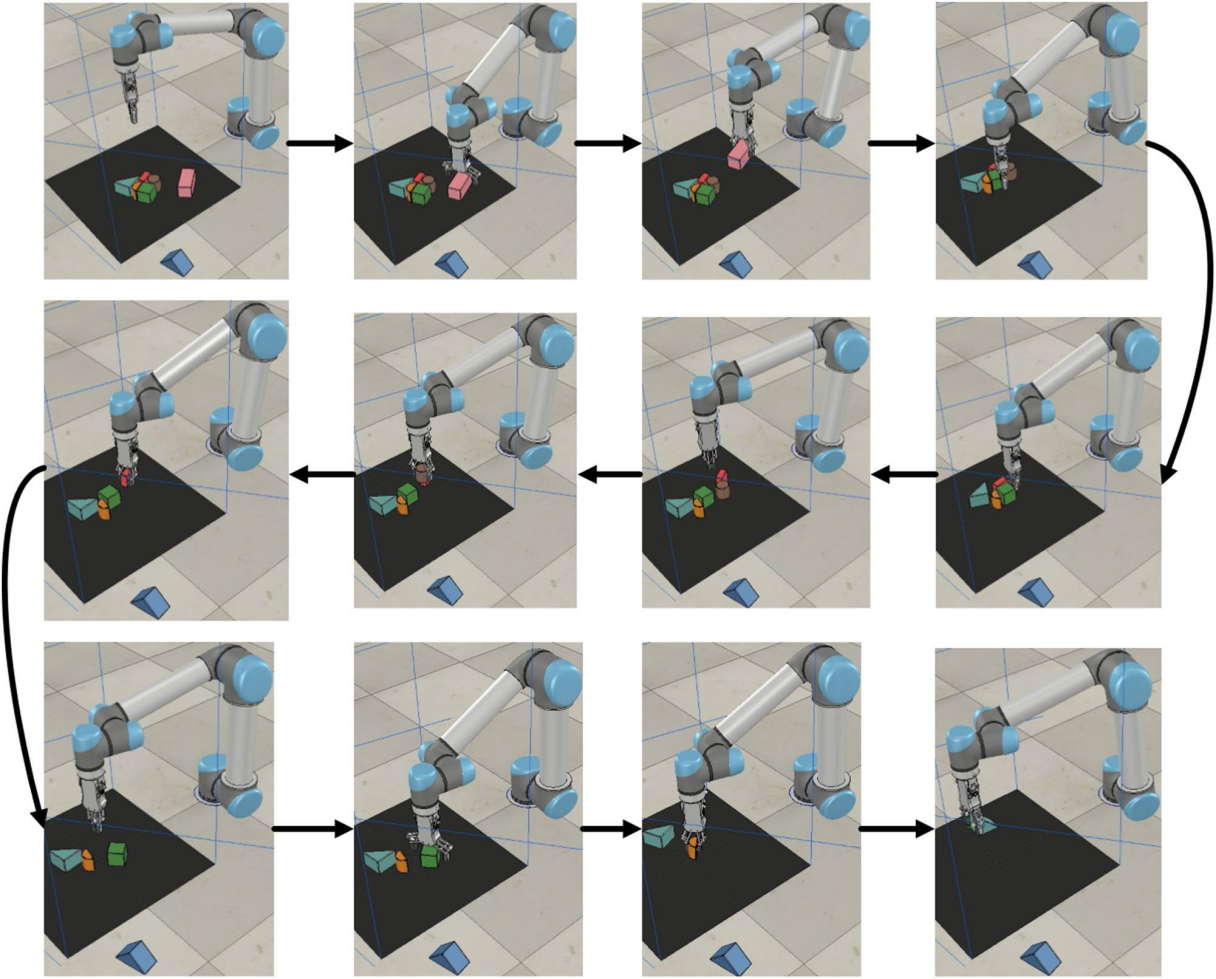
Grasping of unknown objects.

After the model is trained, in order to verify that the pushing-grasping strategy based on DQN and dual viewpoints has a better generalization ability than the grasping strategy based on DQN and single viewpoint, three unknown objects are selected to design three groups of experiments, and three unknown objects which are triangles, semicircles, and cylinders. The settings of scene for the three experiments are as follows: ➀ unknown object + 5 known objects placed in the working space; ➂ unknown objects + 3 known objects placed in the working space; ➄ unknown objects + 1 known object placed in the working space. Use trained model above to make the manipulator to grasp objects in the workspace until the objects in the workspace are grasped successfully or the objects are out of the workspace. In each group of the experiments, each unknown object needs to appear 30 times in total for the convenience of statistics. The experiment in the simulation environment is shown in Fig. 6.

The results of the success rate of grasping with the two methods are shown in Table 1, where U means unknown objects, K means known objects exist in training set.

**Table 1:** Sucess rate of grasping unknown objects.

Test group	Triangle	Semicircle	Cylinder
1U+5K(Our Method)	11/30	14/30	17/30
1U+5K(Grasp Only)	3/30	7/30	6/30
3U+3K(Our Method)	6/30	10/30	14/30
3U+3K(Grasp Only)	2/30	4/30	6/30
5U+1K(Our Method)	5/30	6/30	12/30
5U+1K(Grasp Only)	1/30	2/30	6/30

From data in Table 1, it’s obvious that the DQN-based grasping strategy has a low success rate of grasping when grasping unknown objects, while the pushing-grasping strategy based on DQN and dual viewpoints has a better generalization ability when grasping unknown objects, with a higher success rate especially in grasping cylinders which are similar to the objects in training set, but it should be noted that when there are an increasing number of unknown objects, the success rate of grasping unknown objects is also getting lower. It’s probably for the reason that the arrangement structure of unknown objects is different from the arrangement structure of the objects in the training set.

## Conclusion

In order to solve the problems that robots can’t change the order of objects in complex scenes in intelligent manufacturing, which leads to low success rate of grasping, insufficient information utilization and slow network convergence speed of deep reinforcement learning based grasping methods, a pushing-grasping collaborative sorting method based on DQN and dual viewpoints is proposed in this paper. This strategy obtains the top views of the objects’ point cloud from two viewpoints, combining pushing and grasping actions, with a piecewise reward function, and finally trains to obtain a robot grasping model iteratively, which gives the best action taken by manipulator. Through comparative experiments in V-REP simulation environment, it can be seen from the results that the method proposed in this paper can not only speed up the convergence of the network, but also disrupt the original arrangement structure of the objects by pushing actions, which improves the success rate of grasping obviously, which can come up to 83.5%. And it also has a certain generalization ability when grasping unknown objects.
